# Phenotypic and Genotypic Characterization of *Staphylococcus aureus* Strains from Italian Dairy Products

**DOI:** 10.1155/2009/501362

**Published:** 2010-01-27

**Authors:** Stefano Morandi, Milena Brasca, Cristian Andrighetto, Angiolella Lombardi, Roberta Lodi

**Affiliations:** ^1^CNR Istituto di Scienze delle Produzioni Alimentari (ISPA), Sez. Milano, Via Celoria 2, 20133 Milan, Italy; ^2^Veneto Agricoltura, Istituto per la Qualità e le Tecnologie Agroalimentari, Via San Gaetano 74, 36016 Thiene (VI), Italy

## Abstract

*Staphylococcus aureus* is a known major cause of foodborne illnesses, and milk and dairy products are often contaminated by enterotoxigenic strains of this bacterium. In the present study, 122 *S. aureus* isolates collected from different dairy products were characterised by phenotypic properties, by the distribution of genes encoding staphylococcal enterotoxins (*sea*, *sec*, *sed*, *seg*, *seh*, *sei*, *sej*, and *sel*) and by randomly amplified polymorphic DNA PCR (RAPD-PCR). Moreover, strain resistance to vancomycin and methicillin (oxacillin) was studied. The differences in the RAPD-PCR profiles obtained with the primers M13 and AP4 revealed the presence of a great genetic heterogeneity among the different *S. aureus* strains. Using the primer AP4 and M13, eight groups were distinguished by RAPD-PCR cluster analysis, although, except in few cases, it was not possible to correlate the isolates of different animal species (cow or ovine) with the presence of *se* genes. None of the isolates showed resistance to vancomycin or methicillin.

## 1. Introduction

*Staphylococcus aureus* is an important food-borne pathogen involved in a variety of invasive diseases. Of particular relevance is the ability of some *S. aureus* strains to produce heat stable enterotoxins that cause staphylococcal food poisoning, which ranks as one of the most prevalent worldwide causes of gastroenteritis [1]. 

Eleven major antigenic types of SEs have been recognised (SEA to SEJ) and their corresponding genes have been reported [2]. Recently, other SE toxins were identified (SEK, SEL, SEM, SEN, SEO, and SEU) and the corresponding genes (*se*) described [3–5], but their role in food poisoning is not clear. 

*S. aureus* can gain access to milk either by direct excretion from udders with clinical and subclinical staphylococcal mastitis or by environmental contamination during the handling and processing of raw milk [6, 7].

*S. aureus* is also a frequent cause of human infections which can become especially serious if induced by strains resistant to antimicrobial drugs [8]. In fact, nowadays, antimicrobial resistance has become a major public health problem in many countries due to the constant circulation of resistant bacterial strains in the environment and possible foodstuff contamination. Indeed, it has already been suggested by several authors that the administration of antibiotics to food-producing animals, for therapeutic purposes or as growth promoters, could be a primary selection factor for antimicrobial-resistant bacterial pathogens. Furthermore, *S. aureus* has been reported to frequently show multiple antimicrobial resistance patterns, particularly to methicillin and vancomycin [9, 10].

Several molecular typing methods have been described in order to obtain an accurate and rapid characterization of *S. aureus* isolates, such as coagulase (*coa*) or protein A (*spa*) restriction fragment length polymorphism (RFLP), Multiple-Locus Variable-Number Tandem-Repeat (MLVA), Pulsed-Field Gel Electrophoresis (PFGE), Multilocus Sequence Typing (MLST), and amplified fragment length polymorphism (AFLP). Random amplification of polymorphic DNA (RAPD PCR) has been applied extensively to distinguish different isolates of *S. aureus* [11, 12]. However, there is little information on the RAPD method for typing *S. aureus* strains isolated from dairy products.

In the present study *S. aureus* strains isolated from different dairy products, collected in various Italian regions, were identified at the species level and characterised at the genetic level by means of RAPD-PCR. The isolates were also evaluated for the presence of enterotoxin genes (*sea*, *sec, sed*, *seg*, *seh*, *sei*, *sej,* and *sel*) and for phenotypic activities such as the presence of coagulase, thermonuclease, and hemolytic activity. In addition, the *S. aureus* strains were tested for resistance to methicillin and vancomycin.

## 2. Material and Methods

### 2.1. Source of Bacterial Isolates and S. aureus Identification

The study employed a total of 122 *S. aureus* strains. All the isolates were obtained from the ISPA (Institute of Science of Food Production) bacterial collection and came from different raw milk products (milk, curd, cheeses, butter, and whey) from different Italian regions and animal species. As reported in Table 1, 81 isolates originated from cow, 22 from goat, 17 from sheep, and 2 from buffalo.

A miniaturized biochemical system (Biolog GP Microplate, Biolog, Inc., Hayward, CA, USA) was used to confirm the staphylococcal species. The strains were maintained and propagated in Brain Heart Infusion broth (Oxoid, Milan, Italy) and incubated at 37°C overnight.

Strain identification was also confirmed by *S. aureus* specific primers for the 23S rRNA gene according to Cremonesi [13].

### 2.2. DNA Extraction and Detection of se Genes by Multiplex PCR

DNA was extracted, as described by Cremonesi [14], using one millilitre of the culture incubated in BHI broth overnight at 37°C, containing approximately 1 − 3 × 109 cells. In parallel, cell numbers were verified by total sample counts, following the ISO 6888 1/2:1999 procedure with Baird Parker RPF agar plate [15]. As several studies have described that none of the investigated strains isolated from bovine and goat milk, and related dairy products, harbour any of the *seb, see,* and *sek* genes, s*e* genes, including *sea*, *sec*, *sed*, *seg*, *seh*, *sei*, *sej,* and *sel* were detected by multiplex PCR assay as described by Cremonesi [13]. This PCR assay also included species-specific primers for 23S rRNA, coagulase, and thermonuclease. The reference strains ATCC 700699 (harbouring *sea*, *sec*, *seg,* and *sej* genes), ATCC 23235 (*sed*, *seg*, *sei,* and *sej*), and ATCC 19095 (*sec*, *seh*, *seg* and *sei*) were included as positive controls for the PCR assay.

### 2.3. Investigation of the Phenotypes

The *S. aureus* strains were phenotyped by appraising the heat stable nuclease (TNase) test using Toluidine blu agar (Oxoid) according to ISO 8870:2006 [16] and coagulase determination according to ISO 6888 1/2:1999 [15].

### 2.4. Hemolysis on Blood Agar and Antibiotic Resistance

Hemolytic activity was determined on blood agar (defibrinated sheep blood) (Merck, Darmstad, Germany) at 37°C for 24 hours. The type of hemolysis was recorded as *α*-, *β*-, and double (*α* + *β*). 

Antibiotic susceptibility was determined by the standardized agar diffusion test on Muller-Hinton (Biolife, Milan Italy) using the following disks: vancomycin bioDisc VA30 (30 *μ*g/disk) and methicillin (oxacillin) OX1 (1 *μ*g/disk) (bioMérieux, RCS Lyon, France) according to manufacturer instructions. *S. aureus* ATCC 29213 was used as the reference strain [17]. Isolates were categorized as susceptible and resistant based upon interpretative criteria developed by the National Committee of Clinical Laboratory Standards [18].

### 2.5. RAPD-PCR

RAPD-PCR reactions were performed with primers M13 and AP4. The amplification conditions, as well as electrophoresis and analysis of the amplification products, were the same as those described by Andrighetto [19], except for the amplification cycle of primer AP4 that was modified as follows: an initial step of 95°C for 90  seconds, followed by 35 cycles of 95°C for 30  seconds, 36°C for 60  seconds, and 72°C for 90  seconds. Grouping of the RAPD-PCR profiles was obtained with the Gel Compar 4.1 software package (Applied Maths, Kortrjik, Belgium), using the Pearson product moment correlation coefficient and UPGMA cluster analysis.

## 3. Results and Discussion

### 3.1. Identification of Microbial Isolates from Dairy Products

All 122 isolates were identified by PCR reaction as belonging to *Staphylococcus aureus.* The Biolog GP identification for 27 strains gave different identifications; 2 strains resulted *S. delphini,* 1 *S. xylosus, *1* S. intermedius*, and 1 *S. haemolyticus*; 1 was not identified, and for 21 strains the identification was only at the genus level (*Staphylococcus* spp.). The use of Biolog GP allowed the correct identification of 78% of *S. aureus* isolates, while for the remaining 22% of isolates, species-specific PCR was necessary. All 122 cultures were positive for the presence of coagulase and heat stable nuclease.

### 3.2. Hemolysis Patterns of the S. aureus Isolates

All the tested *S. aureus* presented hemolysis on blood agar plates; 66 strains (54%) showed *β*-hemolysis, 49 (40%) double hemolysis (*α* + *β*), and 7 (6%) *α*-hemolysis. The majority of strains isolated from cow dairy products showed a prevalence of *β*-hemolysis (62%) while 29 strains (36%) gave double hemolysis. *α*-hemolysis was detected in only 2 cow isolates. *β*-hemolysis prevalence in bovine *S. aureus* strains is in full agreement with other research papers [20, 21], but contrary to what was shown in studies conducted by Stephan [22] who, in Switzerland, found double hemolysis in 23 of 34 *S. aureus* isolated from cow milk samples. Most of the *S. aureus* strains derived from goat dairy products (64%) showed double hemolysis, and in none of the isolates *α*− hemolysis was detected. For the strains isolated from sheep dairy products, there was the homogeneous distribution of *α*, *β*, and double-hemolysis (5, 6, and 6 strains). The two strains isolated from buffalo dairy products were *β*-hemolytic on blood agar.

### 3.3. Prevalence of the se Genes in the S. aureus Isolates

The frequency of the *se* genes and the relation between enterotoxins and sample origin are reported in Table 2. Of the 122 *S. aureus* isolates tested, 79 (65%) were found to be positive for one or more *se* gene. The most frequent gene was *sed* (n: 40) followed by *sea*, *sej*, *sec*, *sel,* and *sei*. The gene *seh* was the least frequent. The genes *sec*-*sel* (n: 16) were, in all cases, associated, but only one strain carried them with other genes. In the same way *sej* was always found in combination with *sed*, but *sed* was not necessarily always associated with *sej.* The most frequent *se* gene profiles were *sec*-*sel* (n: 15), *sea*-*sed*-*sej* (n: 14), *sed*-*sej* (n: 13), and *sea* alone (n: 13). Twenty-one *S. aureus* possessed only one type of toxin gene (13 *sea*, 3 *sed*, 1 *seg*, 3 *seh,* and 1 *sei*), while the remaining 58 strains harboured more than one toxin gene. Only 3 isolates harboured *seg* and *sei* that are comprised by the enterotoxin gene cluster (*egc*) [23].

The novel *se* genes (*seg*, *seh*, *sei*, *sej,* and *sel*) were often associated with the classical genes, except for 8 strains that were positive for only one of the newly described *se* or, in some cases, for just a few of them. From the multiplex PCR analysis it appears that there is a certain degree of heterogeneity among the *se* gene profiles; in fact it was possible to group them into 17 gene combinations. 

Comparing the data relative to the strains isolated from cow, goat, sheep, and water buffalo dairy products, 58 of the 81 (72%) strains from cow were positive for *se*, and *sea*, *sed,* and *sej* were found more frequently. Only 2 strains (isolated from Trentino Alto Adige milk and Veneto cheese, two regions of North Italy) were found to have the *sec* gene. Twelve of the 22 *S. aureus* (55%) isolated from goat dairy products harboured *se* genes, and the enterotoxins *sec* and *sel* predominated, each being found in 7 strains. A similar toxin pattern was noted in *S. aureus* isolated from sheep. In fact 53% of the isolates produced enterotoxins, and *sec* and *sel* were the most widespread. The two strains isolated from buffalo did not produce staphylococcal enterotoxins.

This work shows that the *sea* and *sed* genes are dominant and are often associated with *sej* in *S. aureus* isolates. *Sed* and *sej* genes have been localized in the same plasmid [24].

The predominance of enterotoxins A and D contradicts reports from countries such as Brazil, Norway, France, and Japan [25–29], where enterotoxin C *S. aureus* producers were frequently isolated from milk and raw milk cheeses. However, Normanno [10] showed that in Italian dairy products most of the isolated strains produced SED, followed by SEA, SEC, and SEB; moreover in South Korea and in France the *sea* gene was dominant in strains linked to staphylococcal food poisoning studied from 1981 to 2002 [30, 31].

### 3.4. Antibiotic Resistance Profile of the Isolates

All the *S. aureus* strains studied were tested for resistance to antibiotics. The antibiotics selected for the study were vancomycin and methicillin, these being commonly used in the medical and veterinary fields. Of the 122 strains studied 120 were sensitive to vancomycin while the other 2 strains (1 from cow and 1 from sheep isolates) showed, according to NCCLS, intermediate resistance to this antibiotic. None of the strains isolated from dairy products showed resistance to methicillin.

### 3.5. RAPD-PCR Analysis of the Isolates

All 122 isolates considered in this study were characterized by means of RAPD-PCR, a technique used by many to type *S. aureus* isolated from different foodstuffs implicated in staphylococcal food poisoning [32–35], from individual quarter milk and human samples [36–40] and from mastitis milk samples [41]. The RAPD-PCR analyses on all the isolates were carried out with the primers M13 and AP4. The reproducibility value of the RAPD-PCR assay, calculated on the repetition of independent amplifications of *S. aureus* strains, was higher than 95% for both the M13 and AP4 primers. 

**Figure 1:**
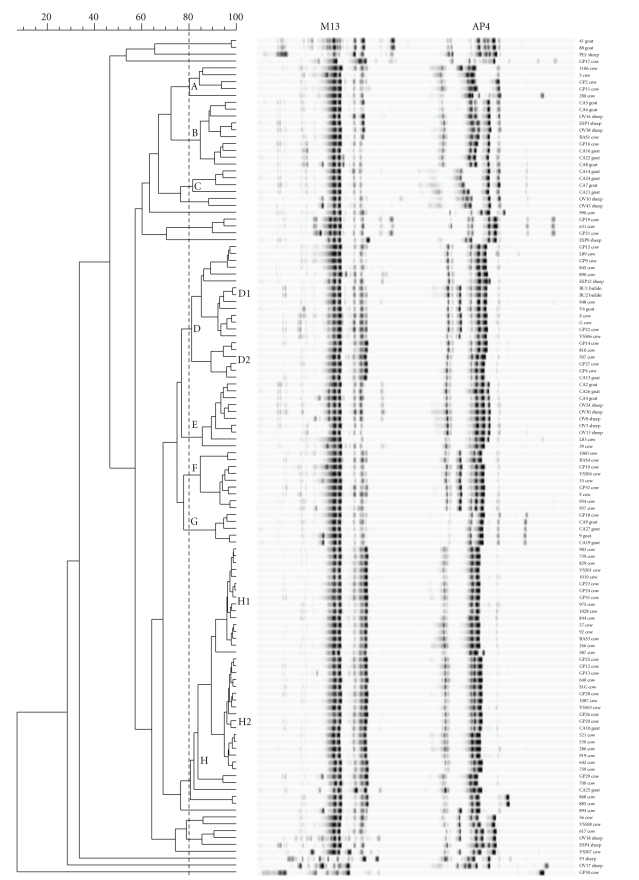
Dendrogram derived from the RAPD-PCR profiles generated with primers M13 and AP4.

Genomic variability in the *S. aureus* strains became evident in the RAPD-PCR analysis (Figure 1). At 80% similarity, 8 distinct clusters were detected. Cluster A grouped 5 *S. aureus* strains isolated from cow dairy products: 4 of the 5 showed the presence of enterotoxin genes and 3 showed *β*-hemolytic activity. Most of the strains grouped in cluster B were isolated from ovine dairy products; this cluster contained 5 strains that came from goat, 3 from sheep, and 2 from cow. Six *S. aureus* isolates were not able to produce enterotoxins and 7 strains showed double hemolysis, 1 *β* and 2 *α*-hemolysis. Cluster C grouped 4 goat isolates, of which 2 strains harboured *sec*-*sel* and 2 were not enterotoxin producers. Double hemolysis was detected in 3 out of 4 strains. Cluster D contained 20 isolates (15 from cow, 2 from goat, 1 from sheep and 2 from water buffalo) and *β*- and double hemolysis were predominant, respectively, in 11 and 8 strains. Within this cluster, only one *S. aureus* strain isolated from sheep showed *α*-hemolysis. Cluster D can be divided into two subclusters (D1 and D2); D1 contained 14 strains (10 from cow, 1 from goat, 1 from sheep and 2 from water buffalo) of which 7 are not toxin producers, while the D2 subcluster grouped 6 isolates (5 from cow and 1 from goat) that all harboured the *sed* gene. Cluster E contained 8 strains that came from ovine dairy products (3 from goat and 5 from sheep) and 2 from cow. The 8 ovine strains showed the presence of enterotoxin genes, 7 harboured *sec*-*sel* and 1 *seg*-*sei*. The 2 strains isolated from cow were not enterotoxin producers. In cluster E *β*-hemolysis was predominant (7/10). All the strains belonging to cluster F were isolated from cow isolates, 6 out of 9 strains were not able to produce enterotoxins, and 5 isolates showed *β*-hemolysis. Cluster G grouped 5 strains (1 from cow and 4 from goat). All isolates were *β*-hemolytic and did not show the presence of enterotoxin genes. Cluster H contained 38 isolates (36 from cow and 2 from goat), and in this cluster we identified two subclusters, H1 (16 isolates) and H2 (17 isolates), characterized by a similarity coefficient of 90%. The 16 cow isolates belonging to the H1 subcluster showed the presence of enterotoxin genes (except one *S. aureus* strain), the strains harbouring singly, or in association with others,* sea* (9), *sed* (15), and *sej *(8), while of the strains grouped in the H2 subcluster (16 cow and 1 goat isolate) 14 showed the presence of the *sea* gene, 11 the *sed,* and 9 the *sej*. In H1 and H2 the *β*-hemolytic isolates predominated, respectively, 16 and 10 strains. Applying an 80% similarity value, 21 *S. aureus* isolates did not enter the 8 clusters.

The RAPD-PCR technique was shown to be efficient in typing the studied strains. The use of the primers allowed the subdivision of the isolates into eight major clusters within which, in some cases, the identified strains had similar characteristics (presence/absence of genes encoding enterotoxins, hemolysis type). As reported by other authors, the results of our research indicate that the presence of toxin genes is not associated with particular RAPD-PCR patterns [12, 42]. In addition, the RAPD-PCR and analyses of the genes encoding for the toxins showed no correlation with the geographical area of origin, whilst in many cases there was correlation with animal species. 

With regard to resistance to antibiotics (vancomycin and methicillin), none of the strains isolated from the dairy products showed resistance, while a low frequency was reported by Normanno et al. [43] who found 3.75% of *S. aureus* resistant to methicillin. Indeed, also enterococci have shown similar results, different authors [44–46] having demonstrated that, in the dairy sector, most strains are sensitive to antibiotics.

## 4. Conclusions

The data acquired in the present work confirm the wide phenotype and genotype diversity of *S. aureus* from dairy products but such diversity was not always able to be intercorrelated. Furthermore, a similar enterotoxin strain incidence was confirmed in isolates from animals suffering mastitis [47]. It is interesting to note, however, that there was no evident correlation between the observed strain variability and the region from which the isolates originated.

## Figures and Tables

**Table 1:** Origin of the 122 *S. aureus* strains examined in this study.

Sample origin	Source	No strains	Regions
			
Cow (*81 strains*)	Raw milk	29	Lombardia
		16	Piemonte
		5	Emilia Romagna
		3	Veneto
		2	Valle d'Aosta
		1	Trentino Alto Adige
		1	Liguria
		1	Puglia
		1	Calabria
	Curd	6	Lombardia
		3	Piemonte
	Cheese	4	Lombardia
		3	Veneto
	Butter	5	Lombardia
		1	Trentino Alto Adige
			
Goat (*22 strains*)	Raw milk	17	Lombardia
	Cheese	5	Lombardia
			
Sheep (*17 strains*)	Raw milk	11	Sardegna
		2	Sicilia
		1	Toscana
	Curd	1	Sicilia
	Cheese	1	Sicilia
	Whey	1	Sicilia
			
Water buffalo	Raw Milk	2	Lazio
(*2 strains*)			

**Table 2:** Degree of heterogeneity among the se gene profiles of *S. aureus*.

*se* gene	Strains	Origin of isolates			
		Cow	Goat	Sheep	Water buffalo
Not detected	43	23	10	8	2
*a*	13	11	2		
*ad*	6	6			
*adj*	14	13	1		
*adgj*	1	1			
*adgij*	1	1			
*ag*	1	1			
*agi*	1		1		
*aghi*	1		1		
*cl*	15	1	7	7	
*cdjl*	1	1			
*d*	3	3			
*dj*	13	13			
*djg*	1	1			
*g*	1			1	
*gi*	3	2		1	
*h*	3	3			
*i*	1	1			
					
Total	122	81	22	17	2
